# Double aortic arch: a comparison of fetal cardiovascular magnetic resonance, postnatal computed tomography and surgical findings

**DOI:** 10.1016/j.jocmr.2024.101053

**Published:** 2024-07-01

**Authors:** Milou P.M. van Poppel, David F.A. Lloyd, Johannes K. Steinweg, Sujeev Mathur, James Wong, Vita Zidere, Simone Speggiorin, Haran Jogeesvaran, Reza Razavi, John M. Simpson, Kuberan Pushparajah, Trisha V. Vigneswaran

**Affiliations:** a School of Biomedical Engineering & Imaging Sciences, King’s College London, King’s Health Partners, St Thomas’ Hospital, London SE1 7EH, UK; b Department of Congenital Heart Disease, Evelina London Children’s Hospital, Guy’s & St Thomas’ NHS Trust, Westminster Bridge Road, London SE1 7EH, UK; c Department of Radiology, Evelina London Children’s Hospital, Guy’s & St Thomas’ NHS Trust, Westminster Bridge Road, London SE1 7EH, UK

**Keywords:** Congenital heart disease, Vascular ring, Prenatal diagnosis, Magnetic resonance imaging, Computed tomography

## Abstract

**Background:**

In double aortic arch (DAA), one of the arches can demonstrate atretic portions postnatally, leading to diagnostic uncertainty due to overlap with isolated right aortic arch (RAA) variants. The main objective of this study is to demonstrate the morphological evolution of different DAA phenotypes from prenatal to postnatal life using three-dimensional (3D) fetal cardiac magnetic resonance (CMR) imaging and postnatal computed tomography (CT)/CMR imaging.

**Methods:**

Three-dimensional fetal CMR was undertaken in fetuses with suspected DAA over a 6-year period (January 2016–January 2022). All cases with surgical confirmation of DAA were retrospectively studied and morphology on fetal CMR was compared to postnatal CT/CMR and surgical findings.

**Results:**

Thirty-four fetuses with surgically confirmed DAA underwent fetal CMR. The RAA was dominant in 32/34 (94%). Postnatal CT/CMR was undertaken at a median age of 3.3 months (interquartile range 2.0–3.9) demonstrating DAA with patency of both arches in 10/34 (29%), with 7 showing signs of coarctation of the left aortic arch (LAA). The LAA isthmus was not present on CT/CMR in 22/34 (65%), and the transverse arch between left carotid and left subclavian artery was not present in 2 cases.

**Conclusion:**

Fetal CMR provides novel insights into perinatal evolution of DAA. The smaller LAA can develop coarctation or atresia related to postnatal constriction of the arterial duct, making diagnosis of DAA challenging with contrast-enhanced CT/CMR. This highlights the potentially important role for prenatal 3D vascular imaging and might improve the interpretation of postnatal imaging.

## Graphical abstract

**Figure:**
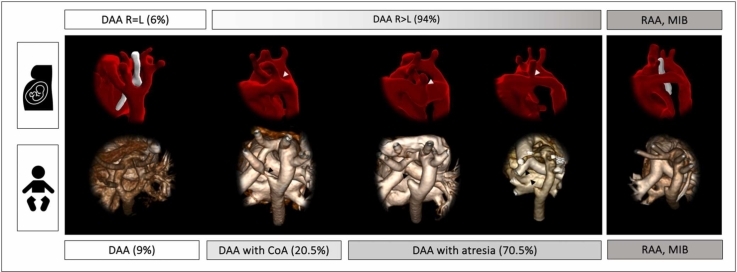

## 1 Introduction

The prenatal diagnosis of vascular rings continues to increase due to the widespread incorporation of the three vessel and tracheal view into routine prenatal ultrasound screening [1], and more cases are identified in-utero than in postnatal life [2], [3], [4], [5]. Various arch abnormalities can produce a vascular ring. A double aortic arch (DAA; where both right aortic arch [RAA] and left aortic arch [LAA] are present), is the form most likely to require postnatal intervention, with one in five displaying symptoms within 24 hours of birth which increases to 45% at 3 months of age [6]. Prenatal diagnosis allows preparation for the possibility of early respiratory difficulty after birth, organization of the appropriate site of delivery, optimization of timing of intervention, and improves clinical outcome after timely surgical relief of the vascular ring [2], [6].

Recent data have shown that there is often significant hypoplasia of one arch (usually the left) of the DAA, which can make it difficult to visualize using prenatal ultrasound, so that some fetuses may be diagnosed with a single RAA [7], [8]. Similarly in postnatal life, the hypoplastic arch can become atretic, again leading to significant uncertainty and/or missed diagnoses using computed tomography (CT) or cardiac magnetic resonance (CMR) imaging [9], [10], [11]. The hypothetical variations of DAA were well-described by Edwards in 1948 [12] and demonstrated in vivo in the surgical literature [13], [14], [15], [16], [17], [18], [19], [20], [21], [22]. While it has been suggested that DAA with a region of atresia of the left arch is a rare variant, recent reports suggest it is more common than previously reported [6]. Development of fetal CMR imaging using motion-correction techniques [23] allows detailed three-dimensional (3D) assessment of suspected DAA in-utero and particularly, can visualize both the right and left aortic isthmuses of a DAA [10], [23]. The primary objective of this study is to compare the 3D fetal CMR appearances in DAA with postnatal CT/CMR to gain potential insights into the morphology and evolution of DAA from fetal life to infancy, particularly the impact of the closure of a fetal shunt, the arterial duct.

## 2 Methods

### 2.1 Patients

All children with surgically confirmed DAA who underwent fetal CMR over a 6-year period (January 2016–January 2022) were identified and retrospectively reviewed from the departmental vascular ring and fetal CMR databases. Patients with major congenital heart disease were excluded.

### 2.2 Clinical pathways

The initial diagnosis was made by the fetal cardiologist using fetal echocardiography and referrals were made for fetal CMR referrals at the discretion of the fetal cardiologist and required consent from the expectant woman. All patients with a prenatally diagnosed DAA or RAA are followed up in our pediatric cardiology department in the first months after birth. Patients were put forward for division of the DAA after a multidisciplinary discussion of clinical presentation, fetal imaging, postnatal imaging, and bronchoscopic findings.

### 2.3 Fetal CMR imaging protocols

Expectant mothers were offered fetal CMR examination in the third trimester of pregnancy. Multiple T2-weighted “black-blood” sequences (standard single-shot turbo spin echo [SSH-TSE]) were acquired on a 1.5T Ingenia magnetic resonance imaging (MRI) system (Philips, Best, the Netherlands). The two-dimensional (2D) data were subsequently processed using an in-house developed and validated motion-correction algorithm (slice-to-volume registration), producing a high-resolution 3D volume (range 0.60–0.85 mm isotropic) allowing detailed visualization of the vasculature in the fetal thorax without the use of intravenous contrast or sedation [23]. Semi-automatic segmentation of the fetal heart was undertaken using ITK-SNAP (version 3.6.0, www.itksnap.org) [45] at the time of clinical reporting to obtain a 3D model. These fetal CMR studies were performed as part of an ethically approved research program and/or a clinical fetal CMR service and all were reported by a pediatric cardiology team subspecialized in fetal and pediatric CMR and reports archived contemporaneously before delivery of the baby.

### 2.4 Pediatric CT/CMR imaging

Contrast-enhanced CT imaging using a SOMATOM Force Dual Source CT scanner (Siemens Healthineers, Erlangen, Germany) or CMR using a 1.5T MRI scanner (Magnetom Aera, Siemens Healthineers, Erlangen, Germany) was performed as part of the clinical assessment in early childhood according to our institutional pathway and reported contemporaneously by a pediatric radiologist or cardiologist [5], [6], [24]. Imaging was reviewed using Sectra PACS IDS7 (Sectra AB, Linköping, Sweden). As part of our institutional pathway, postnatal CT was preferred over CMR at the preference of the respiratory physicians and radiologists that assessment of the lung parenchyma could be incorporated [6], [24]. Some earlier cases were assessed by CMR to confirm vascular anatomy only.

### 2.5 Assessment of vascular morphology

All fetal CMR and postnatal CT/CMR imaging were retrospectively reviewed for the purpose of this study (M.v.P.). The fetal arch anatomy was compared to postnatal CT or CMR and surgical findings. The morphology of the aortic arches was assessed according to the following features: the relative size of the left and RAA; the presence of three components of the aortic arch; retro-esophageal diverticulum arising from the descending aorta; the position of the aortic isthmus; and appearance/morphology of the arches and aortic branching as described previously [11], [25], [26], [27], [28], [29], [30], [31], [32], [33]. The arch components comprised the proximal arch (proximal to the origin of the common carotid artery [CCA]), the transverse arch (between the CCA and subclavian artery [SA]), and the distal arch (between the SA and descending aorta). The term aortic isthmus was used to describe the region between the SA and the insertion of the arterial duct. The term coarctation was used when there was a discrete narrowing of a region, but patency of the vessel was maintained as evidenced by contrast filling the vessel on CT/CMR. Atresia of a segment was defined by the absence of luminal filling with contrast on postnatal CT/CMR.

### 2.6 Ethical approval

Written informed consent allowing fetal CMR research was obtained as part of either the “Intelligent Fetal Imaging and Diagnosis Project-2” (Research Ethics Committee (REC):14/LO/1806) or “Quantification of fetal growth and development using MRI” (REC: 07/H0707/105). Review of the clinical data was approved through the Institutional Clinical Governance Department at Guy’s & St Thomas’ NHS Foundation Trust Hospitals (no: 13556).

## 3 Results

A DAA was confirmed at surgery in 49 cases during the study period of which 34 (69%) had a fetal CMR examination; 32 cases were diagnosed with DAA prenatally by fetal CMR. Two cases were diagnosed prenatally by fetal CMR to have a RAA with left arterial duct and aberrant left SA. The surgical diagnosis was DAA with atresia of the transverse LAA in one patient and DAA with severe transverse LAA hypoplasia in the other. There were no cases with a fetal CMR diagnosis of DAA where a DAA was not confirmed or refuted during surgery.

Fetal CMR examinations were conducted in the third trimester of pregnancy (median gestational age 31.4 weeks, interquartile range [IQR] 30.6–31.8). A complete DAA (proximal arch, transverse arch, distal arch of right arch and proximal arch, transverse arch, and aortic isthmus of left arch) was seen on fetal CMR in 32/34 fetuses. In 2 cases, the LAA was not seen, the fetal CMR dataset was limited in one case due to fetal motion and in the other the LAA was not visualized. The arterial duct was left-sided in all cases. Postnatal contrast-enhanced CT (33/34) or CMR (1/34) was performed in all cases at a median age of 3.3 months (IQR 2.0–3.9). The arterial duct was closed in all cases at the time of postnatal CT/CMR. Surgical relief of the DAA was undertaken at a median age of 5.1 months (IQR 3.6–8.1) via a left posterolateral thoracotomy in 33/34 and via a median sternotomy in one (for concurrent ventricular septal defect closure). Two infants with prenatally diagnosed DAA had postnatal CT appearances strongly suggestive of isolated RAA, leading to a postnatal CT diagnosis (independent of fetal imaging and before multidisciplinary review) of RAA with mirror image branching pattern in one and RAA with aberrant left subclavian artery (ALSA) in another patient. However, both cases were confirmed to be DAA at surgery, with atresia of the LAA isthmus and atresia of the transverse LAA, respectively.

**Fig. 1:**
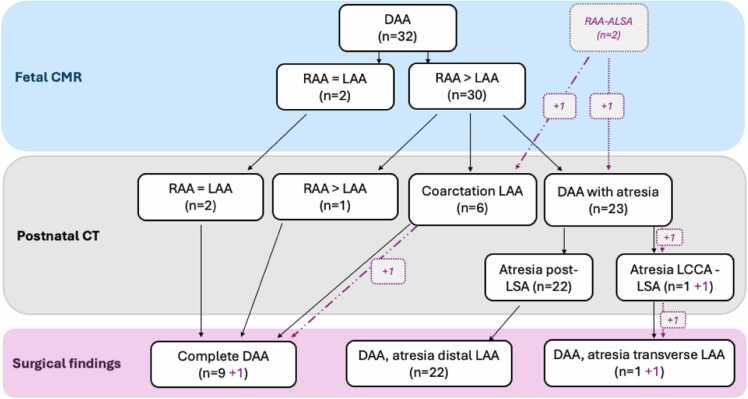
**Flowchart of DAA evolution.** Flowchart demonstrating the evolution and variation of prenatal morphology (as seen on fetal CMR) and postnatal arch morphology (CT/CMR and surgery) in our cohort (n = 34) of DAA. Dashed box and lines represent two cases with fetal CMR diagnosis of RAA but surgical diagnosis of DAA. *ALSA* aberrant left subclavian artery, *CCA* common carotid artery, *DAA* double aortic arch, *LAA* left aortic arch, *RAA* right aortic arch, *SA* subclavian artery, *CMR* cardiovascular magnetic resonance, *CT* computed tomography, *LCCA* left common carotid artery, *LSA* left subclavian artery.

The observed evolution of DAA phenotypes from prenatal to postnatal findings is summarized in Fig. 1 and according to the postnatal morphology in the subsections below.

### 3.1 DAA with balanced arches

**Fig. 2:**
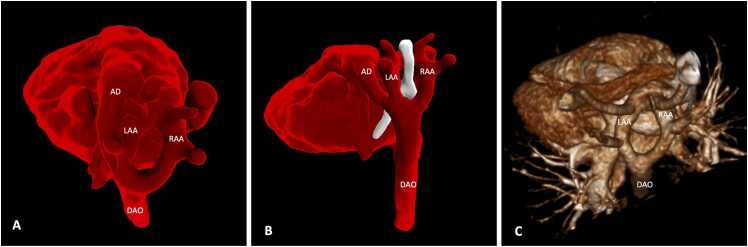
**DAA with balanced arches.** Segmentation of motion-corrected black-blood fetal CMR data (A, B) at 30 weeks gestation shows a complete DAA with similar size left and right aortic arch encircling trachea (white mesh) and esophagus. The LAA courses posteriorly to join the descending aorta. The left-sided arterial duct can also be appreciated inferior to the LAA. Postnatal contrast-enhanced CT (C) at 3 months shows balanced aortic arches. The left arterial duct is no longer patent and therefore not visible on contrast-enhanced CT. *AD* arterial duct, *DAO* descending aorta, *RAA* right aortic arch, *LAA* left aortic arch, *DAA* double aortic arch, *CMR* cardiovascular magnetic resonance, *CT* computed tomography.

There were two fetuses with a complete DAA and equal-sized left and right aortic arches whose anatomy was similar on postnatal CT. Prenatal and postnatal imaging is demonstrated for one of these cases in Fig. 2. In both cases, surgical division of both the RAA and left ductal ligament was undertaken.

### 3.2 DAA with asymmetrical arches

In the majority of patients (30/34), 3D fetal CMR demonstrated a DAA with asymmetrical aortic arches, the RAA was the dominant arch, and all three components of the smaller left arch were patent. In 2/34 patients, only the RAA and left-sided arterial duct were seen on fetal CMR. Significant variations were present in the postnatal imaging in this cohort. Review of postnatal CT/CMR demonstrated a DAA with dominant RAA and smaller LAA in 1/34, LAA coarctation in 7/34, the LAA isthmus was not present on the contrast-enhanced images in 22/34, and the transverse segment of the LAA was not present in 2/34.

#### 3.2.1 Hypoplasia/coarctation of distal LAA

**Fig. 3:**
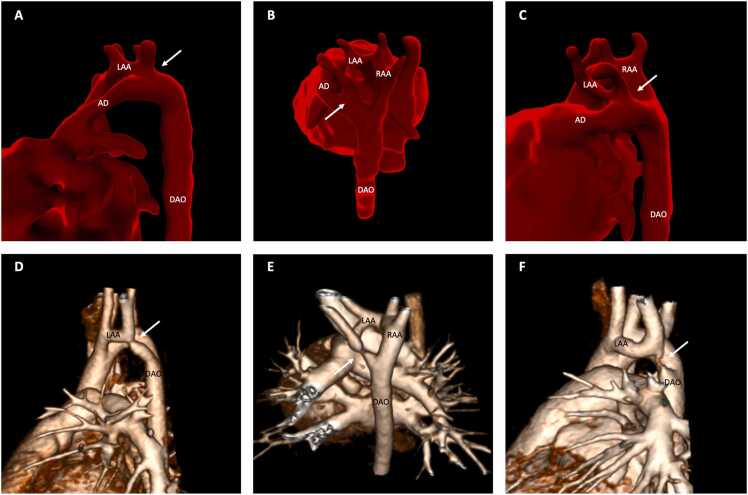
**DAA with asymmetrical arches and postnatal coarctation of smaller arch.** Third-trimester fetal CMR (A-C) showing the LAA coursing posterior to the trachea but with a degree of anterior displacement of the left aortic isthmus (arrowed) before joining the left-sided arterial duct/descending aorta. Corresponding contrast-enhanced CT demonstrated discrete coarctation (D and E) or severe hypoplasia (F) with luminal continuity of the left aortic isthmus (arrowed). *AD* arterial duct, *DAO* descending aorta, *RAA* right aortic arch, *LAA* left aortic arch, *DAA* double aortic arch, *CMR* cardiovasuclar magnetic resonance, *CT* computed tomography.

Of the seven patients with LAA coarctation, two showed long-segment coarctation/severe hypoplasia of the LAA isthmus on CT and five had a discrete region of coarctation (Fig. 3). In all cases, luminal continuity of the LAA isthmus was evident by contrast filling of the vessel on CT. On fetal CMR, the LAA could be seen coursing posterior to the trachea in 6/7 cases, and the LAA isthmus was present in 6/7 but with a degree of anterior displacement in most cases (Fig. 3A and C] before joining the left-sided arterial duct/descending aorta. In one case, the LAA was not seen on fetal CMR which was of limited quality due to excessive motion artifact.

#### 3.2.2 Atresia of distal LAA

**Fig. 4:**
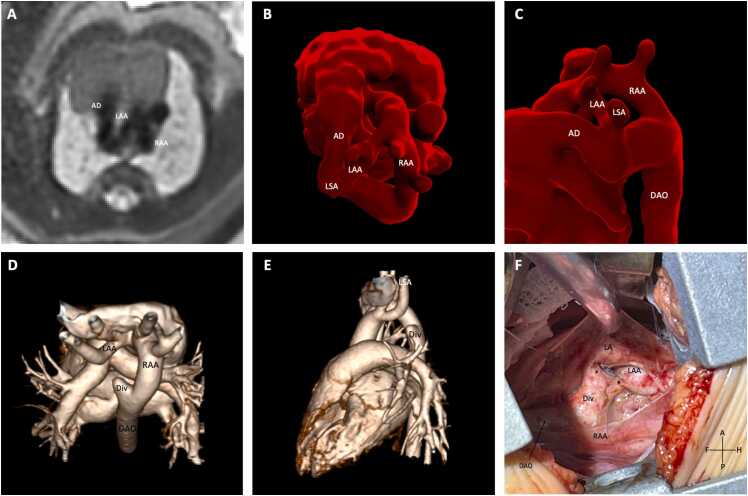
**DAA with LAA isthmal atresia.** Fetal CMR at 34 weeks (A) demonstrates the Z-sign on a transverse plane of the upper mediastinum, formed by a dominant RAA, smaller LAA, and left-sided AD which is a similar view to that seen on fetal echocardiography. The 3D mesh shows a tortuous left-sided AD and apparent complete DAA but with significant anterior displacement of LAA isthmus as demonstrated in superior (B) and left lateral views (C). Postnatal CT (D and E) at 4 months shows an incomplete DAA with LAA isthmal atresia distal to the left SA. Posterior course of the left arch, including tethering of left SA (E), four-vessel sign (D), and diverticulum arising from descending aorta (D), can be identified. Surgery at 9 months (F) demonstrated a (ligamentous) connection (asterisk) between left SA and descending aorta. *AD* arterial duct, *DAO* descending aorta, *Div* diverticulum, *LA* ligamentum arteriosum, *LAA* left aortic arch, *LSA* left subclavian artery, *RAA* right aortic arch, *DAA* double aortic arch, *CMR* cardiovascular magnetic resonance, *CT* computed tomography, *3D* three-dimensional.

**Fig. 5:**
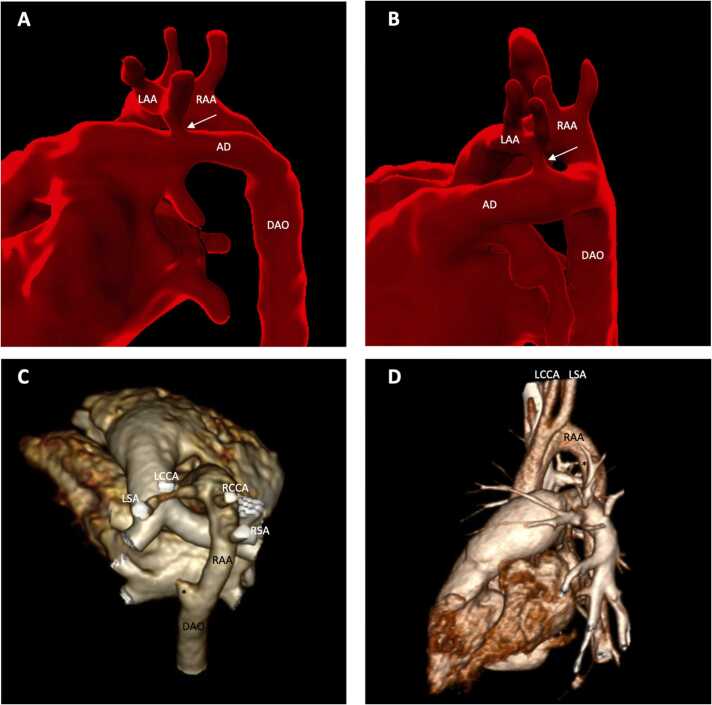
**DAA with LAA isthmal atresia—CT suggestive of RAA.** Visualization of LAA isthmus can be limited on 2D fetal echocardiography when there is a complex spatial relationship or superior-inferior pathway and insertion of the aortic isthmus into arterial duct and severe displacement of the isthmus (arrow A and B). On postnatal CT, the diagnosis appears to be of a single RAA with mirror image branching for both patients (A/C and B/D). The postero-inferior course of the LAA and four-vessel sign are not present (C and D). The clue to the presence of an atretic LAA is the diverticulum on the DAO (*). Both infants were symptomatic and had moderate to severe airway compression diagnosed on bronchoscopy. In both, surgery confirmed the presence of an atretic LAA in addition to the presence of a ligamentum arteriosum. *AD* arterial duct, *DAO* descending aorta, *LAA* left aortic arch, *LCCA* left common carotid artery, *LSA* left subclavian artery, *RAA* right aortic arch, *RCCA* right common carotid artery, *RSA* right subclavian artery, *DAA* double aortic arch, *CT* computed tomography, *2D* two-dimensional.

There were 22 cases where the LAA isthmus was not present on postnatal CT/CMR and acquired atresia of the LAA isthmus was suspected because in all cases, fetal CMR showed a patent LAA isthmus ( Figs. 4 and 5). The fetal CMR findings were confirmed intra-operatively, as an atretic LAA isthmus in addition to the ductal ligament was present. A degree of anterior displacement of the LAA isthmus was seen in all patients on fetal CMR. In four cases, the LAA isthmus passed in a cranio-caudal direction connecting from the underside of the LAA to the superior aspect of the arterial duct creating the distinct appearance of a “coarctation shelf” (Fig. 5A and B). The classical postnatal CT/CMR imaging signs of a DAA such as posterior course of the LAA with tethering/inferior course of the left SA and four-vessel sign could be identified in 18 and 20 cases, respectively (Fig. 4D and E) but were often subtle and absent in the remaining cases—to the degree where RAA with mirror image branching pattern could be suspected in the absence of prenatal imaging (Fig. 5C and D). In all cases, a retro-esophageal aortic diverticulum of varying size could be seen arising from the left side of the descending aorta on postnatal CT/CMR.

#### 3.2.3 Atresia of transverse LAA

This was present in two infants, in one of these fetuses patency of the transverse LAA was noted with fetal CMR examination, and in the other case only the RAA (with left duct and ALSA) was noted on fetal CMR. Contrast-enhanced postnatal CT in both demonstrated only the RAA as postnatally the transverse LAA was no longer visible. In addition, the origins of the SA and CCA were asymmetrical with the left SA arising posterior to the trachea, consistent with a diagnosis of RAA with ALSA. However, intra-operative anatomy demonstrated DAA with atresia of the transverse LAA, in keeping with the fetal CMR findings. In both cases, the transverse LAA, left-sided arterial ductal ligament, and left SA were divided during surgery to release the vascular ring.

## 4 Discussion

### 4.1 Main findings of the study

This study compares 3D fetal CMR to postnatal cross-sectional imaging and surgical findings, describing the evolution of the morphology of various DAA phenotypes from fetal to post-transitional circulation. In all cases, the three components of both arches were patent in-utero, but there were important postnatal changes in the LAA morphology with many cases developing either coarctation of the smaller (left) arch (21%) or atresia of the LAA isthmus (65%) following closure of the arterial duct after birth. In agreement with other studies, the presence of a descending aortic diverticulum on postnatal CT/CMR of a RAA indicates the presence of an additional, atretic left arch.

### 4.2 Physiological interpretation of findings

The concept of selective atresia of part of the aortic arch has been described in the hypothetical DAA model [12] where Edwards proposed the DAA as a basic pattern, representing a (“growth”) adjustment to the classic Rathke model (six arches). The development of atresia of one aortic arch has been previously demonstrated in case reports with fetal echocardiography [9] and also with fetal CMR from our institution [10], [23]; however, this is the first series to investigate and report this in a larger cohort of third-trimester fetuses with DAA and demonstrate that it is a common phenomenon in the setting of a DAA. Using these methods, we have been able to demonstrate that in the presence of a left-sided arterial ductal ligament, isthmal hypoplasia of the left arch can range from discrete coarctation to complete atresia, with the latter being particularly difficult to detect using postnatal CT and easily mistaken for more benign variants of RAA. Left arch coarctation or atresia invariably developed in the region of the arterial ductal ligament, which was also left-sided in all cases in our series. Thus, we postulate that coarctation or atresia of the LAA is likely related to physiological postnatal constriction of the duct. When coarctation or atresia of the left aortic isthmus was present on the postnatal CT, it corresponded to a small LAA isthmus on fetal CMR with a degree of isthmus displacement in most—similar to the “coarctation shelf” seen in coarctation when there is a single aortic arch [34]. Thus, supporting the theory that the atresia/coarctation that is seen after birth in the distal LAA is due to ductal constriction and not an in-utero phenomenon.

### 4.3 Implications for clinical practice

There are a number of vascular abnormalities affecting the position of the aortic arch and its branches which can be diagnosed prenatally and this study has specifically assessed those with a DAA. The precise diagnosis between DAA and RAA with left arterial duct can be challenging on fetal echocardiography with many specialist fetal cardiologists reporting a lack of confidence in differentiating the two on fetal echocardiography [35]. Accurate diagnosis can be achieved with a systematic review [7], but ambiguous cases remain. The current study has provided a greater understanding of the 3D morphology of a DAA which might be useful to understand fetal echocardiographic findings in cases of a suspected DAA particularly avoiding reliance on visualization of the left aortic isthmus to confirm the diagnosis with fetal echocardiography. Accurate differentiation between the various types of vascular rings is crucial for perinatal planning. Infants with DAA are commonly symptomatic at birth and therefore prenatal recognition is valuable [2], [6], [36] and it has been shown that timely surgical relief of the vascular ring might improve post-operative outcome [2], [5], [21], [37], [38]. While various fetal echocardiographic features have been explored to differentiate DAA from other types of vascular rings before birth [7], [39], there are important limitations from fetal position or overlying ductal aneurysms which are commonly seen in the third trimester, and in those late presenting cases, fetal CMR may provide further diagnostic clarity.

The diagnosis of DAA with selective atresia may not always be apparent on postnatal echocardiography [11] or postnatal CT/CMR as the obliterated segments are not visible and the classic imaging signs, such as four-vessel view and a posterior course of the left arch and tethering of the left SA, may be subtle or absent. The findings of our study might aid the interpretation of postnatal imaging and therefore direct the surgical approach. A clue to the presence of a DAA postnatally when only a RAA is visualized on imaging is the presence of a diverticulum arising from the left side of the descending aorta, this was seen in all cases where the LAA isthmus was atretic after birth as previously described [10], [13].

Atresia of a segment of a DAA is a common event and therefore it is important for those reporting the postnatal imaging and managing patients that an atretic segment may be present. This atretic segment of the aortic arch remains as a ligamentous connection and thus, completes the DAA. Contrast-enhanced cross-sectional (CT/CMR) imaging relies upon the patency of the vessels to fill with contrast to demonstrate their presence, whereas fetal CMR using black-blood imaging (SSH-TSE) demonstrates the blood pool and patency of vessels without the requirement of contrast. Therefore, if fetal imaging has led tto a suspected DAA, this should be considered in postnatal evaluation.

The less frequent variant of a DAA is when there is transverse arch atresia between the LSA and LCCA and this is difficult to differentiate from RAA-ALSA. There was only one case diagnosed prenatally in our cohort, the hypoplasia of the transverse LAA was seen on the third-trimester fetal CMR and this was evident as atresia on postnatal CT. A further case was identified in our surgical cohort with this anatomy for which fetal CMR did not identify the left arch which might suggest that it was already atretic in the third trimester or that the resolution of fetal CMR limited its visualization. It is likely that those cases of DAA with transverse arch hypoplasia/atresia represent different embryonic or fetal development which we cannot define based on the two cases in this study.

## 5 Limitations

This study was conducted in a center with a high prenatal diagnosis rate of all types of vascular rings [6], [24], [40], with an established pathway for postnatal assessment of all cases which includes specialist review and cross-sectional imaging and therefore is likely to be reflective of the true population with DAA. We acknowledge that we did not encounter any DAA cases with dominant LAA or cases with right-sided coarctation/atresia which has been reported in the postnatal literature [41] and no cases that developed atresia of the LAA proximal to CCA were reported. All cases reported in this cohort were surgically confirmed to have a DAA and it is acknowledged during this period there were a further four cases of DAA which were confirmed with fetal CMR but have not had surgery and therefore might have different postnatal changes than the cases demonstrated in this study.

Fetal CMR is limited by fetal motion and vessel size (the aortic isthmus can be as small as 2 mm), images in this study have been acquired at a resolution of 1.25 × 1.25 × 2.5 mm and were reconstructed to 0.75 mm isotropic (range 0.6–0.85 mm). Thus, a review of small vascular structures coursing in close proximity to each other can be affected by partial volume effects. All fetal CMR imaging in this report was undertaken in the third trimester. Motion-corrected techniques have undergone recent developments correction of rigorous fetal motion [42], [43], [44] and future comparison of second- and third-trimester fetal CMR might provide additional insights due to improved data quality in the face of excessive fetal motion.

## 6 Conclusion

Three-dimensional fetal CMR provides unique insights in the transition from prenatal to postnatal circulation in patients with a DAA which could be used to improve the detection of clinically important aortic arch anomalies. Atresia of the distal LAA in the setting of a DAA appears to be a postnatal phenomenon, likely related to the closure of the arterial duct after birth. The addition of 2D and 3D fetal imaging may be more accurate in defining a DAA than postnatal CT/CMR alone due to the patency of the arterial duct. Postnatal imaging demonstrates a spectrum of morphologies of DAA, which include coarctation or selective atresia of one limb of the DAA. The presence of a descending aortic diverticulum on postnatal CT/CMR of a RAA suggests the presence of an additional, atretic left arch.

## Funding

This work was supported by Wellcome Trust IEH Award [102431] (the iFIND project) and by core funding from the Wellcome/EPSRC Centre for Medical Engineering [WT203148/Z/16/Z]. The research was funded by the National Institute for Health Research (NIHR) Biomedical Research Centre based at Guy’s and St Thomas’ NHS Foundation Trust and King’s College London and supported by the NIHR Clinical Research Facility. The views expressed are those of the author(s) and not necessarily those of the NHS, the NIHR, or the Department of Health and Social Care. D.L. also acknowledges support from the Rothschild Foundation (2020/2017). For the purpose of Open Access, the author has applied a CC BY public copyright license to any Author Accepted Manuscript version arising from this submission.

## Author contributions

**Vita Zidere:** Writing – review and editing, Methodology, Investigation. **Simone Speggiorin:** Writing – review and editing, Methodology, Investigation, Data curation. **Haran Jogeesvaran:** Writing – review and editing, Methodology, Investigation, Formal analysis, Data curation. **Reza Razavi:** Writing – review and editing, Funding acquisition, Conceptualization. **John M. Simpson:** Writing – review and editing, Methodology, Conceptualization. **Kuberan Pushparajah:** Writing – review and editing, Supervision, Methodology, Investigation, Formal analysis, Data curation, Conceptualization. **Trisha Vigneswaran:** Writing – original draft, Supervision, Methodology, Investigation, Formal analysis, Data curation, Conceptualization. **Milou P.M. van Poppel:** Writing – original draft, Visualization, Methodology, Investigation, Formal analysis, Data curation, Conceptualization. **David F.A. Lloyd:** Writing – review and editing, Supervision, Methodology, Investigation, Conceptualization. **Johannes K. Steinweg:** Writing – review and editing, Methodology, Investigation, Data curation. **Sujeev Mathur:** Writing – review and editing, Methodology, Investigation, Data curation. **James Wong:** Writing – review and editing, Methodology, Investigation, Data curation.

## Declaration of competing interests

The authors declare that they have no known competing financial interests or personal relationships that could have appeared to influence the work reported in this paper.
